# KRAS/NRAS/BRAF Mutation Rate in Saudi Academic Hospital Patients With Colorectal Cancer

**DOI:** 10.7759/cureus.24392

**Published:** 2022-04-22

**Authors:** Samah Saharti

**Affiliations:** 1 Department of Pathology, King Abdulaziz University and King Abdulaziz University Hospital, Jeddah, SAU

**Keywords:** colon cancer molecular pathology, arab population, next-generation sequencing (ngs), colorectal cancer (crc), kras/nras/braf mutations

## Abstract

Background: *KRAS/NRAS/BRAF* mutations are prognostic and predictive molecular biomarkers for colorectal cancers (CRCs). CRC has different frequencies in the population for mutations such as *KRAS*,* NRAS*, and *BRAF*. The aim of this study is to verify the frequency of the somatic *KRAS/NRAS/BRAF* mutations in Saudi academic hospital patients diagnosed with CRC and compare it with those estimated at the local and national levels.

Methods: Out of 280 colorectal carcinomas diagnosed between 2018 and 2021 (primary and secondary), 97 (34.6%) were evaluated by Next Generation Sequencing (NGS) for colorectal cancer molecular markers. Four of these failed the PCR amplification, while 93 were successfully tested. *KRAS*, *NRAS,* and *BRAF* mutation rates and clinical pathological characteristics were recorded.

Results: In this retrospective study, almost half of the tested samples were reported to have a clinically significant mutation (46/93 positive calls, while others were triple-negative). We found that the most prevalent mutation in *KRAS* (45.2%) was followed by *NRAS* (2.2%) and *BRAF* (2.2%). *KRAS* p.G12D accounted for the most frequently resulting variant (17/42, 40.5%). Second in ranking is *KRAS* p. G12V (6/42, 14.3%).

Conclusion: This study is the first to describe the frequency of triple mutations in the city of Jeddah. The findings are consistent with previous research conducted in the Middle East and other local Saudi centers.

## Introduction

Colorectal cancer (CRC) incidence has increased due to external risk factors such as diet, obesity, and sedentary lifestyle [1]. It is the third most popular cancer diagnosed in men and the second most popular cancer in women [2]. In parallel to worldwide figures, Arab countries like Saudi Arabia have shown an increase in CRC incidence as well [3]. In 2016, CRC accounted for the most common cancer in Saudi males and the third in Saudi females, following breast and thyroid. The median age at diagnosis was 59 years for male patients and 57 years for female patients. Histologically, most lesions were typical adenocarcinomas, followed by mucinous adenocarcinomas.

Mortality-wise, one of the major reasons for death worldwide is CRC, representing 9.4% of all cancer deaths [1]. While the number of cases in non-metastatic situations has been detected early by screening, addressability, and increased awareness, about 27% of CRC patients in Saudi Arabia were diagnosed at stage IV [4]. As such, in the past 20 years, the focus of the bulk of cancer research has been directed towards advancing therapeutic modalities in the field of metastatic CRC [5,6].

CRC development is mainly driven by genetic mutation and defective cell regulation [7]. Multiple signaling pathways such as RAS-RAF-MAPK, which play an important role in angiogenesis, cell proliferation, and motility, are activated by the accumulation of these mutations, including *KRAS*, *NRAS*, and *BRAF* [8-10].

In the modern age of personalized cancer treatment, the assessment of genetic mutations is a key element. The understanding and predictability of these mutations have revolutionized the treatment of many malignancies recently, improving outcomes as well as patient care [11,12].

The identification of molecular tumor markers predicting the response to treatment is a fundamental element of improved patient management. However, the cost of targeted therapy has been linked to a substantial financial burden for healthcare. Thus, it is extremely important to select appropriate candidates for specific treatment [13].

Biomarker applications such as *KRAS* and *BRAF* for individualized medicine have voiced translation research. A major determinant ​of panitumumab and cetuximab treatment in colorectal cancer is the presence of mutations in the *KRAS* gene [14-16]. At present, one of the most common methods for predicting the response to the EGFR inhibitors is to test for some "activating" mutations of the *KRAS* gene. EGFR inhibitors show minimal effects in tumors harboring *KRAS* mutations.

As far as is known, *BRAF* mutations appear to be associated with a dismal prognosis. The most common *BRAF* alteration (V600E) is responsible for MAPK pathway activation. Multiple tumors harbor *BRAF* oncogenic mutations. The CRC subgroup, which arose from serrated polyps, has a molecular phenotype described by hypermethylation of CpG in known enhancer gene regions and is named as the CpG island methylated phenotype (CIMP). The serrated pathway is led by BRAFV600E-driven hypermethylation [14]. Thus, the risk of subgroup-specific CRC and disease progression is assessed by advancements in epigenetics.

The rate of *KRAS*/*NRAS*/*BRAF* mutations needs to be further defined in Arab patients [15]. The aim of the study is to investigate the frequency of the triple of somatic *KRAS*/*NRAS*/*BRAF* mutations in an academic tertiary hospital in the city of Jeddah, as it has not been described previously. The data can be used as input features for the development and validation of a model for the prediction and visualization of mutations in machine learning algorithms, such as clinical variants and histological parameters.

## Materials and methods

This retrospective-designed study was conducted at King Abdulaziz University Hospital, Jeddah. Out of 280 patients with sporadic CRC diagnosis (primary or secondary) between October 2018 (when the test was first implemented) and September 2021, 97 tumor tissue samples were subjected to TruSight® Tumor 15 Illumina panel with a combined hotspot mutation test using a 15-gene multiplex platform by Next Generation Sequencing (NGS). After obtaining the unit of biomedical research's ethics committee approval, sequencing results were retrieved from the hospital's electronic information system along with their epidemiological data (age, gender, tumor location, stage, and morphology).

For all of these patients, DNA isolation was carried out using formalin-fixed paraffin-embedded tissue (FFPE). All tissue blocks of the FFPE were obtained from the Histopathology Department and the tumor content was thoroughly checked. Five to eight 10-μm sections were used for DNA for the extraction. The Qiagen AllPrep DNA FFPE Kit is isolated with DNA as per instructions from the manufacturer.

## Results

**Figure 1:**
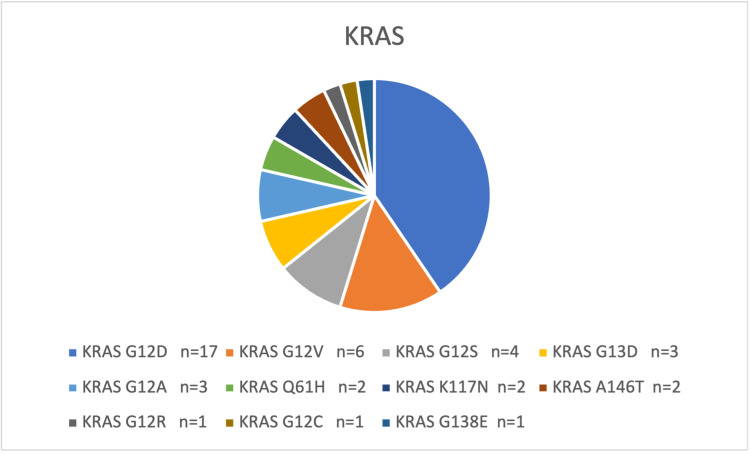
The distribution of KRAS mutations in colorectal cancer.

The mean age of the reported pathogenic variant cases was 57.3, with no gender bias (the male-to-female ratio was almost 1:1). Out of 97 samples, 93 passed the quality parameters. The detected mutations were identified in 46/93 cases (49.5%), while others had triple-negative results. All the studied alterations (*KRAS*/*NRAS*/*BRAF*) were mutually exclusive. The flagged *KRAS* calls were 42/93, representing 45.2%, while *NRAS* and *BRAF* had two detected variants for each, representing 2.2%. The frequency of *KRAS* alterations outweighed *NRAS* and *BRAF*, as illustrated in Table 1. The most common detected *KRAS* oncogenic mutation is G12D, followed by G12V (Table 1). Clinical correlation wise, of all mutated tumors, 30 exhibited aggressive behavior (65.2%) such as recurrence, distant metastasis, and poor response to chemotherapy (27 of which were of *KRAS* pathogenic alterations, 1 *NRAS* and all *BRAF* mutated cases). In addition, 37 positively called samples (73.9%) were left-sided, mostly rectosigmoid (34, 2, 1 mutations found in *KARS*, *NRAS*, and *BRAF,* respectively). Overall, 14 cases (15%) were of the mucinous subtype, most of which (9 cases, 64.3%) carried *KRAS* mutations (Figure 1).

**Table 1:** Mutation frequencies in colorectal cancer.

		Frequency	Percent
KRAS	G12D	17	18.3
	G12V	6	6.5
	G12S	4	4.3
	G13D	3	3.2
	G12A	3	3.2
	Q61H	2	2.2
	K117N	2	2.2
	A146T	2	2.2
	G12R	1	1.1
	G12C	1	1.1
	G138E	1	1.1
NRAS	Q61K	1	1.1
	Q61L	1	1.1
BRAF	V600E	2	2.2
Triple negative		47	50.5
Total		93	100.0

## Discussion

CRC is considered one of the most complex cancers as it is classified according to its pathological features. Several studies have shown interesting results in correlating these features or demographic data with gene mutation. Our figures are similar to those that have been documented globally. The current literature shows that activating *KRAS* mutations predominate in CRC cases (35-45%) with the most frequently detected hotspot codons being 12 and 13 (glycine substituting aspartate p.G12D, p.G13D). In contrast, the mutation rates of *NRAS* are the lowest (1-3%) [16].

At the pathogenesis level, CRC results from traditional pathways (*KRAS*/*BRAF* wild type), alternative pathways (*KRAS* mutation), and the serrated pathways (*BRAF* mutation - dMMR), representing 33.4%, 11.6%, and 0.8% of CRC cases in the Middle East, respectively [17]. Similarly, our CRC cases' molecular analysis revealed that the serrated pathway harboring *BRAF* mutation accounted for a very small percentage (2.2%), which falls behind the adenoma-carcinoma *KRAS*-mutated pathway (45.2%).

The prevalence of CRC drivers’ mutation rates has been slightly variable among different Middle Eastern countries. MD Anderson Cancer Center in Texas published their Arab patients’ data describing that *KRAS*/*NRAS*/*BRAF* prevalence were 44.4%, 4%, and 4%, respectively, compared to 48.4%, 4%, and 4%, respectively, in the matched Western population [18]. Another reference laboratory in the United States has published the *KRAS* mutation frequency in the Middle Eastern countries as follows: Algeria (38.4%), Egypt (27.4%), and Saudi Arabia (34.7%) [19]. In addition, a Lebanese tertiary medical center showed that RAS and *BRAF* mutation rates were 38.5% and 12.9%, respectively [20]. Furthermore, Elbjeirami et al. from Jordan reported the rate of *KRAS* mutations in the Jordanian population with CRC is 44% [21].

Our retrospective study revealed that the *KRAS* mutations are a little higher (45.2%), with left-sided location and disease progression similar to what has been published by other local academic centers. For instance, King Fahad Medical City - Riyadh observed *KRAS* mutations in 42.2% of patients with CRC, mostly p.G12D, p.G12V, and p.G13D. Observed tumors accounted for 51% of the left hemicolon and 23% of the rectum. 74% of the mutations were reported in patients with advanced CRC. 31% of *KRAS*-mutated patients had stage IV disease with wide distant metastasis, particularly liver and lung, compared to 19% in the wild-type group [22]. King Saud bin Abdulaziz University for Health Sciences - Riyadh reported their mutation frequency as follows: *KRAS* (49.6%), *NRAS* (2%), and *BRAF* (0.4%). 44.4% of their patients exhibited wild-type tumors [23]. At Tibah University - Madinah, the proportion of patients with RAS mutations was 43%. 91% of these mutations were in *KRAS*. Codons 12 and 13 were the most detected locations, representing 75% and 20%, respectively, in particular, p.G12D and p.G13D [24]. On follow-up, *KRAS* and *BRAF* mutations predicted poor prognosis and survival [25].

## Conclusions

On the basis of the existence of biomarkers, emerging molecular markers can be a valuable tool for early identification and prevention of CRC, as well as guiding the therapeutic process with individualized therapy. With the rapid advancement of molecular testing and our growing understanding of CRC and its molecular progression, we may soon enter a new era of personalized therapy in which routinely used biomarkers enable more precise patient treatment. In this analysis, *KRAS* p.G12D was the most frequently observed variant associated with CRC in the Saudi population. The second in frequency was *KRAS* p. G12V. Our data are in line with others published locally and regionally. As a result, the current therapeutic option guidelines are applicable to our patients as well. Further research on various populations needs to be conducted in order to obtain a better understanding of their precise position and occurrence.
